# Supplementary material for: Community factors and hospital wide readmission rates: Does context matter?
Source: PLoS One. 2020 Oct 23;15(10):e0240222. doi: 10.1371/journal.pone.0240222 (PMC7584172; doi:10.1371/journal.pone.0240222)
Supplement: Attachment — Submitted filename: HWRcommunity_Submission_FINAL-rev.docx [file pone.0240222.s001.docx]

Title: Community Factors and Hospital Wide Readmission Rates: Does Context Matter?

Authors: Erica S. Spatz,^1,2^ Susannah M. Bernheim,^2,3^ Leora I. Horwitz,^4,5,6^ Jeph Herrin^1^

^1^Section of Cardiovascular Medicine, Yale School of Medicine, New Haven, CT, USA

^2^Yale/Yale New Haven Health Center for Outcomes Research and Evaluation, New Haven, CT, USA

^3^Division of Medicine, Yale School of Medicine, New Haven, CT, USA

^4^Division of Healthcare Delivery Science, Department of Population Health, NYU School of Medicine, New York

^5^Center for Healthcare Innovation and Delivery Science, NYU Langone Health, New York

^6^Division of General Internal Medicine and Clinical Innovation, Department of Medicine, NYU School of Medicine, New York

Conflict of Interest: All authors work under contract with the Centers for Medicare and Medicaid Services (CMS) to develop and maintain performance measures used in public reporting programs.

Funding Support: This study was funded by a grant from the Agency for Healthcare Research and Quality, titled: Understanding Hospital Readmission Rates: Patient, Hospital and Community Effects (1 R01 HS022882 [01](https://nam05.safelinks.protection.outlook.com/?url=https%3A%2F%2Fprojectreporter.nih.gov%2Fproject_info_details.cfm%3Faid%3D8671556%26icde%3D46697278%26ddparam%3D%26ddvalue%3D%26ddsub%3D%26cr%3D1%26csb%3Ddefault%26cs%3DASC%26pball%3D&data=02%7C01%7Cerica.spatz%40yale.edu%7C12e4d0720e5e4b79679508d7420479a5%7Cdd8cbebb21394df8b4114e3e87abeb5c%7C0%7C0%7C637050459310001916&sdata=URFW0R3kdjMVESkmjVIl9IRhC91b4Xk8Ri2bbHg4cbo%3D&reserved=0)).

Corresponding Author:
Erica Spatz, MD

330 Cedar Street

Dana 3 - Section of Cardiovascular Medicine

New Haven, CT 06520

Erica.spatz@yale.edu

Word Count: Abstract 247 words; Manuscript 2,972 words), 17 text pages, 2 tables, 1 figure, 35 references

**ABSTRACT**

**Background**

The environment in which a patient lives influences their health outcomes. However, the degree to which community factors are associated with readmissions is uncertain.

**Objective** To estimate the influence of community factors on the Centers for Medicare & Medicaid Services risk-standardized hospital-wide readmission measure (HWR).

**Research Design** We assessed 71 community variables in 6 domains related to health outcomes: clinical care; health behaviors; social and economic factors; the physical environment; demographics; and social capital.

**Subjects** Medicare fee-for-service patients eligible for the HWR measure between July 2014-June 2015 (n= 6,790,723). Patients were linked to community variables using their 5-digit zip code of residence.

**Methods** We used a random forest algorithm to rank variables for their importance in predicting hospital HWR scores. Variables were entered into 6 domain-specific multivariable regression models in order of decreasing importance. Variables with P-values <0.10 were retained for a final model, after eliminating any that were collinear.

**Results** Among 71 community variables, 19 were retained in the 6 domain models and in the final model. Domains which explained the most to least variance in HWR were: physical environment (R^2^=15%); clinical care (12%); demographics (11%); social and economic environment (7%); health behaviors (9%); and social capital (8%). In the final model, the 19 variables explained more than a quarter of the variance in readmission rate (R^2^=27%).

**Conclusions** Readmissions for a wide range of clinical conditions are influenced by factors relating to the communities in which patients reside. These findings can be used to target efforts to keep patients out of the hospital.

**INTRODUCTION**

Readmission following a hospital discharge represents a potentially avoidable outcome and has been a key focus of recent policy, research and quality improvement efforts.^1-4^ Accordingly, the Centers for Medicare & Medicaid Services (CMS) publicly reports a global measure of readmission following an index hospitalization for virtually any condition (hospital-wide readmission [HWR]).^4^ This measure is risk-adjusted for patient-level clinical factors which influence the risk of readmission. However, social risk factors may also contribute to readmission, both on an individual level, such as individuals’ socioeconomic position, race, ethnicity and culture, gender orientation and sexuality; as well as on a community level, including factors in a patients’ home environment. While the relationship between individual-level social risk factors and outcomes has garnered much attention,^5-9^ less is known about the relative contribution of community-level social, economic and physical factors to outcomes. Communities are multi-dimensional and have attributes that may interact in multiple ways to influence the risk of patients being readmitted soon after discharge.^10-20^ Understanding the relative importance of neighborhood characteristics on individual health outcomes will help policymakers identify and prioritize community-based interventions to improve outcomes that might provide benefits beyond purely hospital-focused activities.

Several community factors may influence a patient’s risk for readmission. For example, some community factors like accessibility of primary care providers, availability of transportation, healthy foods and places to exercise, density of non-profit organizations, and accessible, high-quality acute rehabilitation centers and skilled nursing facilities may directly support or hinder recovery and thus may contribute to patients’ risk of readmission. Additionally, the socioeconomic and social environment, defined by factors such as poverty, unemployment and crime, may exert an indirect effect, as these social ills may result in fewer resources for or attention to health. Assessments of the contribution of these and other community factors to hospital readmissions is important for informing health system and community-based efforts to lower readmission rates.

Accordingly, we adapted and expanded a framework for how community factors relate to health outcomes.^21^ We had 2 primary objectives: to identify variables most associated with hospital readmission rates and to estimate the relative contribution of distinct spheres or domains of influence as well as specific community variables within each domain hypothesized to impact hospital readmission rates. Importantly, and distinct from prior work, we focus on patients admitted for a broad range of different clinical conditions; additionally, we measure the attributes of the communities where patients reside, not where the hospital is located,^18^ which may be more relevant to patient outcomes.^22^ Ultimately, a better understanding of the community factors that have an impact on post-discharge outcomes is essential to promote the development of effective, safe and patient-centered healthcare environments.

**METHODS**

*Overview*

We assessed the contribution of community-level variables to performance on the measure of HWR, a measure of 30-day readmissions among Medicare fee-for-service patients hospitalized for all but a few conditions or procedures. This measure was previously developed by members of our group and is currently publicly-reported by CMS.^4^ We identified a wide range of community variables which we linked to zip code of patient residence, and applied a machine learning approach, random forest, to identify which variables had the most ’importance’ (a parameter assigned by random forest to each variable) for predicting hospital readmission rates. Since variables from different domains may correlate with each other (e.g., poverty and smoking), which may impact statistical significance though not necessarily clinical significance, we first organized all top ranked variables into their respective domains and assessed the relative importance of variables within each domain; the most important variables from each domain were then retained for final comprehensive assessment.

*Conceptual Model*

Our analytic approach was based on a conceptual model we developed by expanding the 4 domains used by the RWJF County Health Rankings framework to include 2 additional domains. The 4 original domains are: clinical care; health behaviors; social and economic factors; and physical environment. The two additional domains are: demographics and social capital. We viewed these 6 domains as representing different ‘spheres’ of influence with different implications for causality and intervention. We hypothesized that factors in each of these domains could have independent influence on the risk of readmission, and that for this reason it would be critical to identify the most important factors related to readmission within each domain, as well as across all domains. Clinical care features of a community can influence readmissions through both quality and access to care, and we included measures of both. Community healthy behaviors measured at the population level capture residents’ healthy activities as well as the availability of open spaces, fresh fruits and vegetables – reflecting the dominant culture towards health and available health resources that may make it more or less easy for a patient returning home from the hospital to engage in health-promoting activities. The social and economic domain includes educational, income, and employment factors which may either directly or indirectly influence patient risk wherein the person has less available resources or the community is deprived of resources which are necessary for recovery and health (e.g., available funds to pay for transportation to appointments; caregiver who can take time off of work; community support groups). The physical environment – including variables such as housing density and air and water quality – is known to affect individual health - impacting respiratory and cardiovascular physiology and increasing people’s risk for admission. To these four domains from the RWJ framework we added two additional community domains which we hypothesized would influence the risk of readmission. One was a demographic domain, which includes age and race/ethnicity characteristics of the community; we hypothesized that communities with greater percentages of minority residents might have more limited access to care or poorer quality of care, beyond what is already captured in the HWR measure, because of historical disparities.^23^ The other additional domain was social capital, which includes metrics on voter turnout and the number of active non-profits and religious organizations;^24^ we hypothesized that communities with greater social cohesion and citizen involvement would also provide additional support to patients who might otherwise return to the hospital.

*Community Factors Data*

The variables for each domain were selected based on the Community Health Rankings, prior literature and/or face validity, and were drawn from several sources, including the U.S. Census Bureau, Bureau of Health Professions Area Resource File (ARF), Northeast Regional Center for Rural Development Social Capital Index, Nursing Home Compare and Home Health Compare.^25,26^ Each variable was assessed at the smallest unit for which there were data, ranging from Census block to county. See Table 1 for a list of the 71 community variables assessed for this analysis, and the level of measurement.

**Table 1**. **Variables included in study, by domain.**

| Domain | Variable | Source | Level | P-value | R2 |
| --- | --- | --- | --- | --- | --- |
| Demographic | |  |  |  |  |
|  | 2011 population estimate Value | RWJ County Health Rankings | FIPS | <0.001 | 0.0355 |
|  | % below 18 years of age | RWJ County Health Rankings | FIPS | <0.001 | 0.0159 |
|  | % aged 65 years and older | RWJ County Health Rankings | FIPS | <0.001 | 0.0050 |
|  | % non-Hispanic African American | RWJ County Health Rankings | FIPS | <0.001 | 0.0801 |
|  | % Am Indian or AK Native | RWJ County Health Rankings | FIPS | <0.001 | 0.0320 |
|  | % Asian | RWJ County Health Rankings | FIPS | <0.001 | 0.0197 |
|  | % Hawaiian/Pacific Islander | RWJ County Health Rankings | FIPS | <0.001 | 0.0301 |
|  | % Hispanic | RWJ County Health Rankings | FIPS | 0.005 | 0.0029 |
|  | % non-Hispanic White | RWJ County Health Rankings | FIPS | <0.001 | 0.0234 |
|  | % non-English Value | RWJ County Health Rankings | FIPS | <0.001 | 0.0122 |
|  |  |  |  |  |  |
| Health Behaviors | |  |  |  |  |
|  | Adult smoking Value | RWJ County Health Rankings | FIPS | <0.001 | 0.0060 |
|  | Adult obesity Value | RWJ County Health Rankings | FIPS | <0.001 | 0.0151 |
|  | Food environment index Value | RWJ County Health Rankings | FIPS | <0.001 | 0.0097 |
|  | Physical inactivity Value | RWJ County Health Rankings | FIPS | <0.001 | 0.0298 |
|  | Access to exercise opportunities Value | RWJ County Health Rankings | FIPS | <0.001 | 0.0511 |
|  | Excessive drinking Value | RWJ County Health Rankings | FIPS | 0.060 | 0.0014 |
|  | Alcohol-impaired driving deaths Value | RWJ County Health Rankings | FIPS | <0.001 | 0.0344 |
|  | Sexually transmitted infections Value | RWJ County Health Rankings | FIPS | <0.001 | 0.0117 |
|  | Teen births Value | RWJ County Health Rankings | FIPS | 0.001 | 0.0037 |
|  | Food insecurity Value | RWJ County Health Rankings | FIPS | <0.001 | 0.0214 |
|  | Limited access to healthy foods Value | RWJ County Health Rankings | FIPS | <0.001 | 0.0181 |
|  | Motor vehicle crash deaths Value | RWJ County Health Rankings | FIPS | <0.001 | 0.0228 |
|  | Drug poisoning deaths Value | RWJ County Health Rankings | FIPS | 0.003 | 0.0033 |
|  |  |  |  |  |  |
| Clinical Care | |  |  |  |  |
|  | Uninsured Value | RWJ County Health Rankings | FIPS | <0.001 | 0.0060 |
|  | Primary care physicians Value | RWJ County Health Rankings | FIPS | 0.026 | 0.0019 |
|  | Dentists Value | RWJ County Health Rankings | FIPS | 0.017 | 0.0021 |
|  | Mental health providers Value | RWJ County Health Rankings | FIPS | 0.954 | -0.0009 |
|  | Diabetic screening Value | RWJ County Health Rankings | FIPS | <0.001 | 0.0078 |
|  | Mammography screening Value | RWJ County Health Rankings | FIPS | <0.001 | 0.0075 |
|  | Nursing Home Quality: Vaccines | Nursing Home Compare | ZIP | <0.001 | 0.0058 |
|  | Nursing Home Quality: Pain | Nursing Home Compare | ZIP | <0.001 | 0.0645 |
|  | HHA Quality: Improvement | Home Health Compare | ZIP | <0.001 | 0.0160 |
|  | HHA Quality: Pain, bedsores | Home Health Compare | ZIP | <0.001 | 0.0072 |
|  | HHA Quality: Vaccines | Home Health Compare | ZIP | <0.001 | 0.0826 |
|  | # Nursing Home beds | Area Health Resource File | FIPS | <0.001 | 0.0444 |
|  | # Hospital Beds | Area Health Resource File | FIPS | <0.001 | 0.0388 |
|  | GPs/100k | Area Health Resource File | FIPS | <0.001 | 0.0669 |
|  | Med Specialists/100k | Area Health Resource File | FIPS | <0.001 | 0.0410 |
|  | Surg Specialists/100k | Area Health Resource File | FIPS | <0.001 | 0.0156 |
|  | Other Specialists/100k | Area Health Resource File | FIPS | <0.001 | 0.0153 |
|  | GPs/Specialists | Area Health Resource File | FIPS | <0.001 | 0.0535 |
|  | # Fed Qual Health Centers | Area Health Resource File | FIPS | <0.001 | 0.0242 |
|  |  |  |  |  |  |
| Social & Economic Environment | |  |  |  |  |
|  | High school graduation Value | RWJ County Health Rankings | FIPS | <0.001 | 0.0238 |
|  | Some college Value | RWJ County Health Rankings | FIPS | 0.020 | 0.0020 |
|  | Unemployment Value | RWJ County Health Rankings | FIPS | <0.001 | 0.0507 |
|  | Children in poverty Value | RWJ County Health Rankings | FIPS | <0.001 | 0.0237 |
|  | Income inequality Value | RWJ County Health Rankings | FIPS | <0.001 | 0.0679 |
|  | Children of single parents Value | RWJ County Health Rankings | FIPS | <0.001 | 0.0397 |
|  | Violent crime Value | RWJ County Health Rankings | FIPS | <0.001 | 0.0392 |
|  | Injury deaths Value | RWJ County Health Rankings | FIPS | <0.001 | 0.0195 |
|  | % Commute car | American Community Survey | FIPS | <0.001 | 0.0345 |
|  | % Commute bike | American Community Survey | FIPS | <0.001 | 0.0144 |
|  | % Commute work at home | American Community Survey | FIPS | <0.001 | 0.0265 |
|  | % Commute public | American Community Survey | FIPS | <0.001 | 0.0316 |
|  | % Preschool | American Community Survey | FIPS | <0.001 | 0.0186 |
|  | % Kindergarten | American Community Survey | FIPS | <0.001 | 0.0342 |
|  | Median Home Value | American Community Survey | FIPS | <0.001 | 0.0060 |
|  |  |  |  |  |  |
| Physical Environment | |  |  |  |  |
|  | Particulate air population Value | RWJ County Health Rankings | FIPS | <0.001 | 0.0224 |
|  | Drinking water violations Value | RWJ County Health Rankings | FIPS | <0.001 | 0.0087 |
|  | Severe housing problems Value | RWJ County Health Rankings | FIPS | <0.001 | 0.0449 |
|  | Driving alone to work Value | RWJ County Health Rankings | FIPS | <0.001 | 0.0181 |
|  | Long commute - driving alone Value | RWJ County Health Rankings | FIPS | <0.001 | 0.0711 |
|  | Housing Density | Area Health Resource File | FIPS | <0.001 | 0.0532 |
|  | Good Air Days | Area Health Resource File | FIPS | 0.001 | 0.0038 |
|  | Daily Fine Particulate Matter | Area Health Resource File | FIPS | <0.001 | 0.0222 |
|  | Toxic Sites | Area Health Resource File | FIPS | <0.001 | 0.0099 |
|  | Housing Units | American Community Survey | FIPS | <0.001 | 0.0413 |
|  |  |  |  |  |  |
| Social Capital | |  |  |  |  |
|  | Social Capital: Associations | Social Capital Index | FIPS | <0.001 | 0.0388 |
|  | Social Capital: Voter turnout | Social Capital Index | FIPS | <0.001 | 0.0277 |
|  | Social Capital: Census response | Social Capital Index | FIPS | <0.001 | 0.0257 |
|  | Social Capital: Non-profits | Social Capital Index | FIPS | <0.001 | 0.0312 |

*All variables were stratified into quintiles

**P-values and R^2^ are from a bivariate regression model, weighted for volume

*Hospital Wide Readmission Measure*

The HWR measure, previously described,^4^ was used as the main outcome for this analysis, since this measure captures the broadest range of patients who are hospitalized and at-risk for readmission. Briefly, the HWR measure is a volume-weighted logarithmic mean of the standardized readmission rates (SRRs) of 5 specialty cohorts: surgical, medical, cardiovascular, cardiorespiratory and neurological. The readmission risk ratio of each cohort is calculated as the ratio of the predicted readmissions divided by the expected readmissions. The numerator is the sum of the predicted probability of readmission for all readmissions, estimated using a hierarchical logistic regression model that includes a hospital-specific effect estimated for each hospital as well as covariates for age, principal diagnosis and comorbidity. The denominator is the same as the numerator but replaces the hospital-specific effect in the model with the average hospital-specific effect of all hospitals in the sample. Only readmissions that are deemed as unplanned are included in the outcome. To exclude planned readmissions, the HWR uses a “planned readmission” algorithm which identifies procedures that are: 1) “always” or “potentially” planned procedures; and 2) not associated with an acute medical discharge diagnosis code; we used version 4.0 of this algorithm.^27^ For analyses we used the logarithmic mean SRR. We used 2015-2016 Medicare Part A to identify the cohort and to assess the outcome for the measurement period July 1, 2015 through June 30, 2016.

*Analysis*

We linked all variables to Medicare FFS patients included in the HWR measure using their 5-digit zip code of residence. For zip codes that crossed more than one county, if only county-level data were available we used the value for the county with the greatest population. For each hospital we then averaged the factor value over all patients discharged from that hospital and categorized these means into quintiles based on their distribution across hospitals. We then summarized hospital HWR SRR for each factor quintile, reporting mean and standard deviation.

Our analytic approach focused on identifying key variables both within each domain and across all domains. Therefore, we used a multi-step analysis. First, because of the large number of candidate community variables and potential for interaction effects, we used a random forest (RF) algorithm to identify the variables with greatest influence on risk standardized readmissions. The RF approach is robust to interactions, missing data, and collinearity, using an ensemble of small classification trees to construct a ‘forest’ which best predicts the outcome.^28^ It is also self-validating in that each tree is trained on a new random half sample and validated on the second half. Our RF incorporated a linear regression model with hospital standardized risk ratio as outcome to classify variables; we constructed 10,000 trees, each using 3 randomly selected variables. The final RF was used to assign an importance score to each variable, representing the proportion of decision paths which ran through that variable.

Though the random forest algorithm can rank variables on importance, it cannot provide estimates of independent effects for each variable. Therefore, in the second step, for each domain we identified the most important factor from the RF results and estimated a domain specific model using that variable. We then sequentially added the next most important variables in that domain, assessing the change in adjusted R^2^, and retaining additional variables that increased the R^2^ by a (relative) 10% or more. The rationale for estimating separate domain models is to identify key variables within each domain which may have less relative importance when assessing effects across all domains. Because variables from different domains might be highly correlated, we assessed the variance decomposition matrix for all retained variables, in order to eliminate any variables that were redundant (as indicated by a condition number of at least 20 and a common variance portion greater than 0.50).^29^ These retained variables were entered into a final multivariable model, which collectively assessed the key variables from each domain for an overall assessment of the relative contribution of community variables to readmission rates. All regression models were hospital level models, weighted for the readmission denominator volume, and for each we report the proportion of variance explained R^2^ and the overall Wald P-value for each variable.

The Institutional Review Boards of Yale School of Medicine and New York University School of Medicine approved the study. All analysis was performed using SAS 9.3 (SAS Institute Inc, Cary NC, USA), Stata 15.1 (StataCorp, College Station TX, USA), R 3.5.2 (R Foundation for Statistical Computing, Vienna, Austria), and the R package *randomForestSRC*.

**RESULTS**

After excluding patients with a planned index admission, who were transferred to another hospital, or who died, enrolled in hospice or left against medical advice during the index admission, the final cohort was 6,790,723 discharges from 4,711 hospitals. The mean (SD) risk standardized readmission rate was 15.3% (0.81%) and the median (interquartile range) was 15.3% (14.8% – 15.8%). Among the 71 community variables, the RF algorithm and sequential model selection identified 19 variables for inclusion in the domain specific models. The 10 most important factors associated with higher readmissions in the random forest model were (in descending order of importance): increasing percent of population that is non-Hispanic African American; lower rate of vaccination by home health agencies; poorer nursing home quality related to pain management; severe housing problems; greater physical inactivity; lower density of social associations; greater income inequality; children living in poverty; lower Census response rates; and greater housing density (Figure 1).
**Figure 1 Caption. Results of random forest analysis, showing relative importance of each factor for predicting hospital overall standardized readmission ratio (SRR).**

The domains that explained the most to least variation were: physical environment (R^2^ for the domain specific model =15%); clinical care (12%); demographic (11%); social and economic environment (7%); health behaviors (9%); and social capital (8%). Of the 19 variables, none were eliminated by the variance decomposition assessment. Thus all 19 variables were included in the final model (Table 2); all variables except voting participation were significant; together these contextual factors explained more than a quarter of the variance in hospital HWR readmission rate (R^2^=27%).

**Table 2.** **Final multivariable model.**

| **Final Model** | |  |  |  |
| --- | --- | --- | --- | --- |
|  |  |  |  |  |
|  |  |  |  |  |
| **Variable** | **Value** | **Mean (SD)** | **P-value** | **R2** |
|  |  |  |  |  |
|  |  |  |  | 0.2658 |
| Housing Density | |  | 0.012 |  |
|  | 1 | ref |  |  |
|  | 2 | -0.202 (0.100) |  |  |
|  | 3 | -0.266 (0.104) |  |  |
|  | 4 | -0.359 (0.111) |  |  |
|  | 5 | -0.296 (0.120) |  |  |
| Daily Fine Particulate Matter | | | <0.001 |  |
|  | 1 | ref |  |  |
|  | 2 | 0.192 (0.056) |  |  |
|  | 3 | 0.159 (0.060) |  |  |
|  | 4 | 0.018 (0.064) |  |  |
|  | 5 | -0.126 (0.066) |  |  |
| # Nursing Home beds | |  | <0.001 |  |
|  | 1 | ref |  |  |
|  | 2 | 0.076 (0.060) |  |  |
|  | 3 | 0.177 (0.060) |  |  |
|  | 4 | 0.074 (0.062) |  |  |
|  | 5 | 0.270 (0.064) |  |  |
| HHA Quality: Vaccines | |  | <0.001 |  |
|  | 1 | ref |  |  |
|  | 2 | -0.153 (0.047) |  |  |
|  | 3 | -0.195 (0.050) |  |  |
|  | 4 | -0.250 (0.056) |  |  |
|  | 5 | -0.333 (0.062) |  |  |
| Nursing Home Quality: Pain | | | 0.004 |  |
|  | 1 | ref |  |  |
|  | 2 | 0.053 (0.049) |  |  |
|  | 3 | -0.097 (0.055) |  |  |
|  | 4 | -0.002 (0.060) |  |  |
|  | 5 | 0.057 (0.069) |  |  |
| % non-Hispanic White | |  | <0.001 |  |
|  | 1 | ref |  |  |
|  | 2 | 0.011 (0.052) |  |  |
|  | 3 | 0.182 (0.064) |  |  |
|  | 4 | 0.293 (0.079) |  |  |
|  | 5 | 0.588 (0.104) |  |  |
| Alcohol-impaired driving deaths | | | <0.001 |  |
|  | 1 | ref |  |  |
|  | 2 | 0.107 (0.049) |  |  |
|  | 3 | 0.067 (0.050) |  |  |
|  | 4 | 0.022 (0.051) |  |  |
|  | 5 | -0.123 (0.057) |  |  |
| Severe housing problems | | | 0.015 |  |
|  | 1 | ref |  |  |
|  | 2 | 0.090 (0.069) |  |  |
|  | 3 | 0.108 (0.077) |  |  |
|  | 4 | 0.238 (0.087) |  |  |
|  | 5 | 0.300 (0.102) |  |  |
| Long commute - driving alone | | | <0.001 |  |
|  | 1 | ref |  |  |
|  | 2 | 0.142 (0.062) |  |  |
|  | 3 | 0.250 (0.065) |  |  |
|  | 4 | 0.344 (0.068) |  |  |
|  | 5 | 0.298 (0.076) |  |  |
| Food insecurity | |  | <0.001 |  |
|  | 1 | ref |  |  |
|  | 2 | -0.175 (0.051) |  |  |
|  | 3 | -0.311 (0.053) |  |  |
|  | 4 | -0.273 (0.059) |  |  |
|  | 5 | -0.067 (0.072) |  |  |
| Income inequality | | | <0.001 |  |
|  | 1 | ref |  |  |
|  | 2 | 0.070 (0.054) |  |  |
|  | 3 | 0.085 (0.057) |  |  |
|  | 4 | 0.265 (0.061) |  |  |
|  | 5 | 0.232 (0.071) |  |  |
| % non-Hispanic African American | | | <0.001 |  |
|  | 1 | ref |  |  |
|  | 2 | 0.159 (0.077) |  |  |
|  | 3 | 0.347 (0.086) |  |  |
|  | 4 | 0.587 (0.095) |  |  |
|  | 5 | 0.537 (0.108) |  |  |
| % American Indian or Alaskan Native | | | 0.014 |  |
|  | 1 | ref |  |  |
|  | 2 | -0.022 (0.048) |  |  |
|  | 3 | -0.149 (0.051) |  |  |
|  | 4 | -0.135 (0.063) |  |  |
|  | 5 | -0.113 (0.076) |  |  |
| Driving alone to work | | | 0.004 |  |
|  | 1 | ref |  |  |
|  | 2 | -0.132 (0.055) |  |  |
|  | 3 | -0.075 (0.062) |  |  |
|  | 4 | -0.038 (0.067) |  |  |
|  | 5 | 0.074 (0.075) |  |  |
| Physical inactivity | | | <0.001 |  |
|  | 1 | ref |  |  |
|  | 2 | 0.219 (0.053) |  |  |
|  | 3 | 0.396 (0.062) |  |  |
|  | 4 | 0.343 (0.076) |  |  |
|  | 5 | 0.434 (0.093) |  |  |
| Adult smoking | |  | 0.004 |  |
|  | 1 | ref |  |  |
|  | 2 | -0.050 (0.051) |  |  |
|  | 3 | 0.080 (0.061) |  |  |
|  | 4 | 0.079 (0.072) |  |  |
|  | 5 | 0.211 (0.084) |  |  |
| Social Capital: Associations | | | <0.001 |  |
|  | 1 | ref |  |  |
|  | 2 | -0.278 (0.049) |  |  |
|  | 3 | -0.460 (0.062) |  |  |
|  | 4 | -0.403 (0.070) |  |  |
|  | 5 | -0.361 (0.088) |  |  |
| Social Capital: Voter turnout | | | 0.354 |  |
|  | 1 | ref |  |  |
|  | 2 | 0.047 (0.051) |  |  |
|  | 3 | 0.007 (0.054) |  |  |
|  | 4 | 0.013 (0.055) |  |  |
|  | 5 | -0.061 (0.062) |  |  |
| Social Capital: Census response | | | 0.046 |  |
|  | 1 | ref |  |  |
|  | 2 | -0.101 (0.064) |  |  |
|  | 3 | 0.018 (0.067) |  |  |
|  | 4 | -0.074 (0.073) |  |  |
|  | 5 | -0.085 (0.084) |  |  |

**DISCUSSION**

In this comprehensive examination of a range of contextual factors and their association with all-cause clinically risk-adjusted hospital readmission rates we found that a range of community variables across 6 distinct domains were independently associated with readmission rates; the 19 community variables which were most associated with readmission rates explained 27% of the variation in hospital-wide readmissions. Importantly, among the variables most accounting for this variation, many could be modifiable by local, state and federal governments, community organizations, hospitals, and health care systems. For example, these findings could give impetus for healthcare delivery systems to partner with community agencies to improve the quality of home care, or to partner with local community health centers to improve vaccination rates. Next, as previously demonstrated, demographics and social and economic factors independently contributed to outcomes, including the proportion of the community that was African American or Native American or Alaskan, as well as the unemployment rate, the level of income inequality and the percent of children living in poverty. These associations amplify known racial and economic disparities that need to be addressed. Finally, we found that housing density and commuting, and people’s social connectedness and engagement were significant. This provides evidence to support the investments several health payors and systems are now making. Payors, such as Medicaid, are increasingly allowing waivers that encourage patients to use premiums towards social issues; hospitals could lobby for such waivers.^30^ Moreover, hospitals are becoming more involved in the composition and quality of the medical community in which their patients live.^21,31,32^ For example, some hospitals are partnering with nursing homes with a track record of high-quality performance and quality improvement initiatives.^33^ Whether these efforts will result in reduced readmission rates should be a focus of future study.

The observation that social risk factors contribute to readmission rates is not new but is also worthy of further attention. As part of its implementation of the IMPACT Act of 2014, ASPE is charged with providing analyses and recommendations on considering social risk within CMS payment programs. Moreover, as of this year, CMS is stratifying HRRP penalties according to the proportion of low SES patients at hospitals. Global measures of community-level SES are being considered in other CMS programs, including the ACO program and the Merit-based Incentive Payment System (MIPS) program. Indeed many ACOs are already working to address community factors such as housing quality and transportation, with the goal of reducing health care costs and improving quality outcomes.^34,35^ Understanding the effects of contextual factors across a range of healthcare environments and settings is important for coordinating efforts to address these factors and reducing readmissions.

Finally, we found that 3 variables indicating communities’ social capital (Census response rates, voter turnout and density of social associations) explained 8% of the variation in readmission rates. Unfortunately, we do not have detailed data available about the types of social supports existing in these communities. However, in extrapolating these findings we might infer that in communities where residents have limited family or friend support, and limited engagement with religious institutions and community centers, there may be a need for hospitals or other health entities to furnish outpatient supports. Patients discharged from the hospital are weak and convalescing – and providing assistance with basic meals and shopping, transportation to appointments, along with emotional support needs to be considered.

These findings have several limitations. Community level variables may not represent the reality of an individual’s living situation or community. We selected the most granular level of variable collection, though we still expect there to be heterogeneity within each unit. Still, these variables are intended to inform a hospital’s readmission rates, and not to predict the readmission rate for any one individual. Having an understanding of the community’s characteristics can inform broader strategies to reduce readmission rates among patients from “vulnerable communities.” Second, we lacked data to fully describe the domains conceptualized as impacting readmission rates. For example, in the domain of social support, several factors known to impact outcomes, including community cohesion and resilience, have not been assessed in this population, which may have underestimated the effect of social support in the model. Third, it is unknown whether these variables would also have the same effect on the readmission rates of other populations, for example, younger or commercially insured patients, or on other outcomes, such as mortality. Finally, we are largely unable to determine the degree to which community factors are contributing above and beyond individual factors because many of these factors (e.g., income, commute to work, health behaviors such as exercise and diet) are not known to us on an individual patient level. As such, it is possible that we are measuring factors that are a proxy for individual risk rather than identifying some intrinsic characteristics of communities that are inherently meaningful. Hopefully, attention to contextual factors will drive more individual and community-level data so that we can best understand patient risk and implement and study strategies to reduce that risk.

**CONCLUSION**

This comprehensive examination of publicly available contextual factors centered on patient residence identified community level variables across a number of domains which explained almost a quarter of variation in hospital-wide readmission rates. These findings provide a number of targets that policymakers, payers, community organizations and hospitals can and should engage with in order to reduce readmission rates, and provide safer, more efficient, patient-centered care.

**ACKNOWLEDGMENTS:** We are grateful for the assistance of Dr. Li Li, PhD, MS – a senior statistician at the Center for Outcomes Research and Evaluation during the time this study was conducted.

References

1.	Desai NR, Ross JS, Kwon JY, et al. Association Between Hospital Penalty Status Under the Hospital Readmission Reduction Program and Readmission Rates for Target and Nontarget Conditions. *JAMA.* 2016;316(24):2647-2656.

2.	Angraal S, Khera R, Zhou S, et al. Trends in 30-Day Readmission Rates for Medicare and Non-Medicare Patients in the Era of the Affordable Care Act. *Am J Med.* 2018;131(11):1324-1331 e1314.

3.	Horwitz LI, Bernheim SM, Ross JS, et al. Hospital Characteristics Associated With Risk-standardized Readmission Rates. *Med Care.* 2017;55(5):528-534.

4.	Horwitz LI, Partovian C, Lin Z, et al. Development and use of an administrative claims measure for profiling hospital-wide performance on 30-day unplanned readmission. *Ann Intern Med.* 2014;161(10 Suppl):S66-75.

5.	Calvillo-King L, Arnold D, Eubank KJ, et al. Impact of social factors on risk of readmission or mortality in pneumonia and heart failure: systematic review. *J Gen Intern Med.* 2013;28(2):269-282.

6.	Krumholz HM, Bernheim SM. Considering the role of socioeconomic status in hospital outcomes measures. *Ann Intern Med.* 2014;161(11):833-834.

7.	Damiani G, Salvatori E, Silvestrini G, et al. Influence of socioeconomic factors on hospital readmissions for heart failure and acute myocardial infarction in patients 65 years and older: evidence from a systematic review. *Clin Interv Aging.* 2015;10:237-245.

8.	Kangovi S, Barg FK, Carter T, et al. Challenges faced by patients with low socioeconomic status during the post-hospital transition. *J Gen Intern Med.* 2014;29(2):283-289.

9.	Kangovi S, Barg FK, Carter T, Long JA, Shannon R, Grande D. Understanding why patients of low socioeconomic status prefer hospitals over ambulatory care. *Health Aff (Millwood).* 2013;32(7):1196-1203.

10.	Brewster AL, Lee S, Curry LA, Bradley EH. Association Between Community Social Capital and Hospital Readmission Rates. *Popul Health Manag.* 2019;22(1):40-47.

11.	Vest JR, Kern LM, Silver MD, Kaushal R, investigators H. The potential for community-based health information exchange systems to reduce hospital readmissions. *J Am Med Inform Assoc.* 2015;22(2):435-442.

12.	McCarthy D, Johnson MB, Audet AM. Recasting readmissions by placing the hospital role in community context. *JAMA.* 2013;309(4):351-352.

13.	Kind AJ, Jencks S, Brock J, et al. Neighborhood socioeconomic disadvantage and 30-day rehospitalization: a retrospective cohort study. *Ann Intern Med.* 2014;161(11):765-774.

14.	Hu J, Gonsahn MD, Nerenz DR. Socioeconomic status and readmissions: evidence from an urban teaching hospital. *Health Aff (Millwood).* 2014;33(5):778-785.

15.	Hu J, Kind AJH, Nerenz D. Area Deprivation Index Predicts Readmission Risk at an Urban Teaching Hospital. *Am J Med Qual.* 2018;33(5):493-501.

16.	Jung D, Kind A, Robert S, Buckingham W, DuGoff E. Linking Neighborhood Context and Health in Community-Dwelling Older Adults in the Medicare Advantage Program. *J Am Geriatr Soc.* 2018;66(6):1158-1164.

17.	Villanueva C, Aggarwal B. The association between neighborhood socioeconomic status and clinical outcomes among patients 1 year after hospitalization for cardiovascular disease. *J Community Health.* 2013;38(4):690-697.

18.	Herrin J, St Andre J, Kenward K, Joshi MS, Audet AM, Hines SC. Community factors and hospital readmission rates. *Health Serv Res.* 2015;50(1):20-39.

19.	Gaskin DJ, Zare H, Vazin R, Love D, Steinwachs D. Racial and Ethnic Composition of Hospitals' Service Areas and the Likelihood of Being Penalized for Excess Readmissions by the Medicare Program. *Med Care.* 2018;56(11):934-943.

20.	Pandolfi MM, Wang Y, Spenard A, et al. Associations between nursing home performance and hospital 30-day readmissions for acute myocardial infarction, heart failure and pneumonia at the healthcare community level in the United States. *Int J Older People Nurs.* 2017;12(4).

21.	Brewster AL, Brault MA, Tan AX, Curry LA, Bradley EH. Patterns of Collaboration among Health Care and Social Services Providers in Communities with Lower Health Care Utilization and Costs. *Health Serv Res.* 2018;53 Suppl 1:2892-2909.

22.	Aswani MS, Kilgore ML, Becker DJ, Redden DT, Sen B, Blackburn J. Differential Impact of Hospital and Community Factors on Medicare Readmission Penalties. *Health Serv Res.* 2018;53(6):4416-4436.

23.	Riley WJ. Health disparities: gaps in access, quality and affordability of medical care. *Trans Am Clin Climatol Assoc.* 2012;123:167-172; discussion 172-164.

24.	Kern LM, Edwards A, Kaushal R. The Patient-Centered Medical Home and Associations With Health Care Quality and Utilization: A 5-Year Cohort StudyThe Patient-Centered Medical Home and Health Care Quality and Utilization. *Annals of Internal Medicine.* 2016;164(6):395-405.

25.	Robert Wood Johnson Foundation County Health Rankings. <http://www.countyhealthrankings.org/>. Accessed May 14, 2014, 2014.

26.	Area Health Resources Files (AHRF) 2013-2014. US Department of Health and Human Services, Health Resources and Services Administration, Bureau of Health Workforce, Rockville, MD.

27.	CMS Planned Readmission Algorithm 4.0 <http://aann.org/uploads/Condition_Specific_Readmission_Measures.pdf>.

28.	Brieman L. Random forests. Mach Learn 2001;45:5-32.

29.	Belsley D, Kuh E, Welsch R. 1980. Regression Diagnostics. Hoboken, NJ. Wiley.

30.	North Carolina Department of Health and Human Services (NCDHHS). Medicaid Expansion. Healthy Opportunities Pilot Overview. <https://www.ncdhhs.gov/about/department-initiatives/healthy-opportunities/healthy-opportunities-pilots/healthy-0> Accessed June 10, 2019.

31.	Hsiao YL, Bass EB, Wu AW, et al. Implementation of a comprehensive program to improve coordination of care in an urban academic health care system. *J Health Organ Manag.* 2018;32(5):638-657.

32.	Berkowitz SA, Parashuram S, Rowan K, et al. Association of a Care Coordination Model With Health Care Costs and Utilization: The Johns Hopkins Community Health Partnership (J-CHiP). *JAMA Netw Open.* 2018;1(7):e184273.

33.	Taylor, M. Healthcare Financial Management Association (HFMA) News. Partnering with Local SNFs Lowers Readmissions: Study. Leavitt Partners. <https://www.hfma.org/Content.aspx?id=54800> Accessed June 10, 2019.

34.	Fisher ES, Corrigan J. Accountable health communities: Getting there from here. *JAMA.* 2014;312(20):2093-2094.

35.	Fraze T, Lewis VA, Rodriguez HP, Fisher ES. Housing, Transportation, And Food: How ACOs Seek To Improve Population Health By Addressing Nonmedical Needs Of Patients. *Health Aff (Millwood).* 2016;35(11):2109-2115.
