# Supplementary material for: Community factors and hospital wide readmission rates: Does context matter?
Source: PLoS One. 2020 Oct 23;15(10):e0240222. doi: 10.1371/journal.pone.0240222 (PMC7584172; doi:10.1371/journal.pone.0240222)
Supplement: Attachment — Submitted filename: Comment - HWR.docx [file pone.0240222.s002.docx]

Comment – HWR

The manuscript is generally scientifically sound with information that will contribute to knowledge and with implications for new strategies in patient management to reduce readmissions. The following observations however need to be corrected.

**Methods**

The study area is left out in the methods. Is the data for the whole of USA?

It is desirable to have examples of such conditions and procedures that were excluded.

**Results**

Figure 1 not provided in this manuscript

**Discussion**

Pg 17 - Cite some previous studies that observed social risk factors contribution to readmission rates. Compare findings of this study with previous studies (if there are). It must be clearly stated if there are no previous studies or similar studies to be compared with this study.
