# Supplementary material for: Community factors and hospital wide readmission rates: Does context matter?
Source: PLoS One. 2020 Oct 23;15(10):e0240222. doi: 10.1371/journal.pone.0240222 (PMC7584172; doi:10.1371/journal.pone.0240222)
Supplement: Attachment — Submitted filename: Response to Reviewers_v3.docx [file pone.0240222.s003.docx]

Thank you for the opportunity to revise this manuscript. We have included a point-by-point response below.

**Editor:**

**Financial Disclosure statement still reads as follows: "This study was funded by a grant from the Agency for Healthcare Research and Quality, titled: Understanding Hospital Readmission Rates: Patient, Hospital and Community Effects (1 R01 HS022882 01)."**

We have added the initials of Authors SMB, LIH, JH, as funded by the above grant.

**Please see an itemized response to the reviewers’ comments:**

**Reviewer #1:**

**Title: Community Factors and Hospital Wide Readmission Rates: Does Context Matter?**

**This manuscript provides valuable information about community factors relationship with hospital-wide readmission. However, it should be revised professionally. Please find my comments in follow:**

**General:
1. The authors should follow the author guideline of PlosOne journal.**

**2. Please edit the manuscript in terms of some grammar errors and Punctuation.**

We appreciate these suggestions, and have made corrections as noted in the tracked changes version.

**3. Abstract:
You should specify where the study was conducted or which country data was used for analysis.
" R2" should be added for each of percentage of domains.**

We added the country in which this study was conducted.

***Objective*** *To estimate the influence of community factors on the Centers for Medicare & Medicaid Services risk-standardized hospital-wide readmission measure (HWR) – a quality performance measure in the U.S.*

We also added R^2^ as suggested.

**4. Introduction:
Page 4, line3: “report”**

Thank you. We have made this edit.

**Paragraph 2 do not have references, please add**
We added the following references:

10.	Brewster AL, Lee S, Curry LA, Bradley EH. Association Between Community Social Capital and Hospital Readmission Rates. *Popul Health Manag.* 2019;22(1):40-47.

13.	Kind AJ, Jencks S, Brock J, et al. Neighborhood socioeconomic disadvantage and 30-day rehospitalization: a retrospective cohort study. *Ann Intern Med.* 2014;161(11):765-774.

15.	Hu J, Kind AJH, Nerenz D. Area Deprivation Index Predicts Readmission Risk at an Urban Teaching Hospital. *Am J Med Qual.* 2018;33(5):493-501.

16.	Jung D, Kind A, Robert S, Buckingham W, DuGoff E. Linking Neighborhood Context and Health in Community-Dwelling Older Adults in the Medicare Advantage Program. *J Am Geriatr Soc.* 2018;66(6):1158-1164.

17.	Villanueva C, Aggarwal B. The association between neighborhood socioeconomic status and clinical outcomes among patients 1 year after hospitalization for cardiovascular disease. *J Community Health.* 2013;38(4):690-697.

18.	Herrin J, St Andre J, Kenward K, Joshi MS, Audet AM, Hines SC. Community factors and hospital readmission rates. *Health Serv Res.* 2015;50(1):20-39.

19.	Gaskin DJ, Zare H, Vazin R, Love D, Steinwachs D. Racial and Ethnic Composition of Hospitals' Service Areas and the Likelihood of Being Penalized for Excess Readmissions by the Medicare Program. *Med Care.* 2018;56(11):934-943.

20.	Pandolfi MM, Wang Y, Spenard A, et al. Associations between nursing home performance and hospital 30-day readmissions for acute myocardial infarction, heart failure and pneumonia at the healthcare community level in the United States. *Int J Older People Nurs.* 2017;12(4).

21.	Brewster AL, Brault MA, Tan AX, Curry LA, Bradley EH. Patterns of Collaboration among Health Care and Social Services Providers in Communities with Lower Health Care Utilization and Costs. *Health Serv Res.* 2018;53 Suppl 1:2892-2909.

**5. Methods:**
**Article writing should be standard to make it easier for readers to follow the article, so make the following changes
Please move the “conceptual model” to the introduction section**

We thank you for this suggestion. We have moved the conceptual model to the introduction with minor modifications. Please see the 3^rd^ paragraph of the introduction.

**6. Then in the methods discuss clearly about: 1. Design and Setting**

We added a Design and Setting section to the beginning of the Methods:

*Design and Setting*

*The context for this study was the United States. Medicare is a federal program, providing insurance coverage to people aged 65 years and older and who are on long-term disability. The Centers for Medicare and Medicaid Services, through with the Medicare program is administered, also generates hospital quality performance metrics which are publicly reported, and which are tied to value-based care programs. We assessed U.S. based community factors, as described below, in relation to a hospital quality measure of readmissions.*

**7. Participants**

*We added this text to clarify the population included in the study:*

*Participants*

*Patients included in the hospital measure of all-cause readmissions are Medicare fee-for-service beneficiaries aged 65 years and older who are hospitalized for any condition other than cancer care, psychiatric conditions, and rehabilitation care for fitting prostheses. Patient who left against medical advice or are transferred to another acute-care setting are excluded. Among these patients, we assessed the hospital risk-standardized readmission rate, as described below. We also assessed patients’ community context, based on the county of their 5-digit zip code; also described in more detail below.*

**8. Study Variables (including Main variable and Confounders)**

New headings were added:

*Independent Study Variables: Community factors*

*Dependent Study Variable: Hospital Wide Readmission Measure*

**9. Please write the full form of the "RWJF" in first use.**

Thank you. This was corrected.

**9. Please develop a section under the subheading of "Ethical Considerations” in the method.
We added the following:**

*Ethical Considerations*

*This study was conducted with de-identified data of the patients and the hospitals and considered exempt from review by the Institutional Review Board.*

**10. Results:
“R2" should be added for each of percentage of domains**

We added R^2^ to each of the percentages.

**11. Table 2: More detail about multivariable model should be added including:**

**1. Describe qualitatively value “1…5”
2. Beta coefficient
3. CI 95%**
We thank the reviewer for this suggestion. We have added a footnote to the table identifying the values 1-5 as representing quintiles of each variable. Table 2 already includes beta coefficients, along with their standard errors. We have chosen not to report 95% confidence intervals because they would represent comparison of each effect relative to the reference group, which we have chosen for convenience to always be the lowest quintile – by reporting standard errors instead, readers can more directly compare coefficients with each other.

**12. Discussion:
Please specify and justified the role of race/ethnicity in HWR in relation to minority diminish return theory (MDR) that recently developed by number of scholars such as DR. Assari and Bazargan in US context.**

Thank you for this suggestion. We agree that MDR is important to mention, and is one of the reasons we sought to add the domain of demographics to the conceptual framework for assessing factors contributing to hospital readmissions.

In the introduction, as part of our conceptual model, we added the following:

*To these four domains from the RWJF County Health Rankings framework we added two additional community domains which we hypothesized would influence the risk of readmission. One was a demographic domain, which includes age and race/ethnicity characteristics of the community; we hypothesized that communities with greater percentages of minority residents have more limited access to care and poorer quality of care, beyond what is already captured in the HWR measure, because of historical disparities.^24^* ***and that other socioeconomic factors (e.g., unemployment; income; education) have different effects in minority populations.^25-27^***

In the discussion, we have added the following:

*Next, as previously demonstrated, demographics and social and economic factors independently contributed to outcomes, including the proportion of the community that was African American or Native American or Alaskan, as well as the unemployment rate, the level of income inequality and the percent of children living in poverty. These associations amplify known racial and economic disparities –* ***each of which needs to be separately considered. While race is associated with lower socioeconomic status, it is also the case that racial minorities derive less benefits from higher socioeconomic status; i.e., racial disparities in health outcomes persist even among high income and highly educated groups.^31-33^ This is known as the Minorities’ Diminished Return (MDR) theory, which can serve as a compass for addressing racial and ethnic disparities in health outcomes.^34^***

**Reviewer #2: The manuscript is generally scientifically sound with information that will contribute to knowledge and with implications for new strategies in patient management to reduce readmissions. The following observations however need to be corrected.**

1. **Methods
   The study area is left out in the methods. Is the data for the whole of USA?**

Thank you. We have added a section entitled, Design and Setting, to the beginning of the Methods.

***Design and Setting***

*The context for this study was the United States. Medicare is a federal program, providing insurance coverage to people aged 65 years and older and who are on long-term disability. The Centers for Medicare and Medicaid Services, through which the Medicare program is administered, also generates hospital quality performance metrics which are publicly reported, and which are tied to value-based care programs. We assessed U.S. based community factors, as described below, in relation to a hospital quality measure of readmissions.*

*Specifically, we assessed the contribution of community-level variables to performance on the measure of HWR, a measure of 30-day readmissions among Medicare fee-for-service patients hospitalized for all but a few conditions (e.g., psychiatric conditions) or procedures that are associated with “planned” or elective admissions described below (e.g., staged percutaneous coronary intervention). This measure was previously developed by members of our group and is currently publicly-reported by CMS^4^ We identified a wide range of community variables which we linked to zip code of patient residence, and applied a machine learning approach, random forest, to identify which variables had the most ’importance’ (a parameter assigned by random forest to each variable) for predicting hospital readmission rates. Since variables from different domains may correlate with each other (e.g., poverty and smoking), which may impact statistical significance though not necessarily clinical significance, we first organized all top ranked variables into their respective domains and assessed the relative importance of variables within each domain; the most important variables from each domain were then retained for final comprehensive assessment.*

2. **It is desirable to have examples of such conditions and procedures that were excluded.**
3. **Results
   Figure 1 not provided in this manuscript**

Thank you, this has been added.

4. **Discussion
   Pg 17 - Cite some previous studies that observed social risk factors contribution to readmission rates. Compare findings of this study with previous studies (if there are). It must be clearly stated if there are no previous studies or similar studies to be compared with this study.**

We have edited the Discussion for flow and have added the following paragraph comparing out study to others.

*The observation that social risk factors contribute to readmission rates is not new, but prior studies have not assessed all cause index admissions, the range of factors explored in this study and the differential effects of their impact on readmission. In a national study of hospital readmission rates, Herrin et al assessed 3 community contextual domains: socioeconomic status; access to care; and nursing home status.^18^ They looked at acute myocardial infarction, heart failure and pneumonia discharges only, and found that 58% of the variation in performance was explained by these factors, with the most potent factor being access to care as measured by number of primary care physicians per capita. Other studies have examined singular domains. For example, a study by Pandolfi et al also found that a community’s nursing home quality is associated with hospital readmission rates^20^ and a study by Brewster et al found that social capital was associated with readmission rates.^10^ Our study attempts to offer a fuller picture of how a community’s well-being can impact health outcomes.*
