# Supplementary material for: Community factors and hospital wide readmission rates: Does context matter?
Source: PLoS One. 2020 Oct 23;15(10):e0240222. doi: 10.1371/journal.pone.0240222 (PMC7584172; doi:10.1371/journal.pone.0240222)
Supplement: Attachment — Submitted filename: Response to Reviewers_v4.docx [file pone.0240222.s004.docx]

Thank you for the opportunity to revise this manuscript. We have included a point-by-point response below.

**Reviewer #1:**

**Thank you for addressing my comments. I did not find a footnote in table 2 identifying the values 1-5 as representing quintiles of each variable that you mentioned in the reviewer response.**

Response: We have updated Table 2 to denote that the values 1-5 are quintiles. This is noted by a Q1-Q5 in the table and with the following footnote:

*Values represent quintiles for each variable

**Reviewer #2:**

**No additional new comment for the authors. Authors have addressed comments raised in initial review earlier submitted**

Response: Thank you
